# CloudDOE: A User-Friendly Tool for Deploying Hadoop Clouds and Analyzing High-Throughput Sequencing Data with MapReduce

**DOI:** 10.1371/journal.pone.0098146

**Published:** 2014-06-04

**Authors:** Wei-Chun Chung, Chien-Chih Chen, Jan-Ming Ho, Chung-Yen Lin, Wen-Lian Hsu, Yu-Chun Wang, D. T. Lee, Feipei Lai, Chih-Wei Huang, Yu-Jung Chang

**Affiliations:** 1 Institute of Information Science, Academia Sinica, Taipei, Taiwan; 2 Department of Computer Science and Information Engineering, National Taiwan University, Taipei, Taiwan; 3 Research Center for Information Technology Innovation, Academia Sinica, Taipei, Taiwan; 4 Department of Computer Science and Information Engineering, National Chung Hsing University, Taichung, Taiwan; CNRS UMR7622 & University Paris 6 Pierre-et-Marie-Curie, France

## Abstract

**Background:**

Explosive growth of next-generation sequencing data has resulted in ultra-large-scale data sets and ensuing computational problems. Cloud computing provides an on-demand and scalable environment for large-scale data analysis. Using a MapReduce framework, data and workload can be distributed via a network to computers in the cloud to substantially reduce computational latency. Hadoop/MapReduce has been successfully adopted in bioinformatics for genome assembly, mapping reads to genomes, and finding single nucleotide polymorphisms. Major cloud providers offer Hadoop cloud services to their users. However, it remains technically challenging to deploy a Hadoop cloud for those who prefer to run MapReduce programs in a cluster without built-in Hadoop/MapReduce.

**Results:**

We present CloudDOE, a platform-independent software package implemented in Java. CloudDOE encapsulates technical details behind a user-friendly graphical interface, thus liberating scientists from having to perform complicated operational procedures. Users are guided through the user interface to deploy a Hadoop cloud within in-house computing environments and to run applications specifically targeted for bioinformatics, including CloudBurst, CloudBrush, and CloudRS. One may also use CloudDOE on top of a public cloud. CloudDOE consists of three wizards, i.e., Deploy, Operate, and Extend wizards. Deploy wizard is designed to aid the system administrator to deploy a Hadoop cloud. It installs Java runtime environment version 1.6 and Hadoop version 0.20.203, and initiates the service automatically. Operate wizard allows the user to run a MapReduce application on the dashboard list. To extend the dashboard list, the administrator may install a new MapReduce application using Extend wizard.

**Conclusions:**

CloudDOE is a user-friendly tool for deploying a Hadoop cloud. Its smart wizards substantially reduce the complexity and costs of deployment, execution, enhancement, and management. Interested users may collaborate to improve the source code of CloudDOE to further incorporate more MapReduce bioinformatics tools into CloudDOE and support next-generation big data open source tools, e.g., Hadoop BigTop and Spark. Availability: CloudDOE is distributed under Apache License 2.0 and is freely available at http://clouddoe.iis.sinica.edu.tw/.

## Introduction

Progress in computer science and technology has vastly promoted the development of genetic research in the past few decades. Next-generation sequencing (NGS) is a particularly notable technology for genetics and computational biology research. The explosive growth of NGS data has already resulted in ultra-large-scale datasets and various computational problems for conventional NGS tools; for instance, insufficient computation resources and undesirably long execution times [1]. To overcome the issues associated with processing of large-scale data, MapReduce [2] and its Java implementation, Hadoop [3], were introduced. MapReduce is a framework that processes huge datasets in parallel by utilizing a large number of computers simultaneously, in which the computing resources can be allocated dynamically. In the programming model of MapReduce, developers only need to focus on implementing their programs by writing appropriate mapper and reducer procedures. Data and computations within the framework are automatically stored and executed across all computers to obtain redundancy, fault tolerance, parallelization, and load balance. Therefore, an increasing number of tools in bioinformatics [1], [4] are successfully being adapted to fit within the MapReduce programming model in order to analyze large biological datasets using cloud computing, e.g., mapping reads to human genomes [5], calculating expression of RNA data [6], finding single nucleotide polymorphisms [7], performing *de novo* genome assembly [8], and achieving error correction of reads [9]. Some bioinformatics tools have also been developed for Hadoop ecosystems [10], [11], [12]. However, usability remains one of the main obstacles for cloud computing [13]. The prerequisite procedures of running MapReduce programs, including deploying Hadoop environments on computer clusters and executing programs through a series of technical Hadoop commands, pose considerable challenges for biological research laboratories that are interested in using MapReduce.

Several existing approaches have been developed in an attempt to ease the burden of deploying and managing a Hadoop cloud. The hicloud-hadoop [14] open-source project focuses on automatically deploying a Hadoop environment on Hinet hicloud [15]. Apache Whirr [16] provides a unified application programming interface for users to initiate cloud services from providers, e.g., Amazon EC2 [17] and Rackspace Cloud Servers [18]. Amazon EMR [19] is a well-known service for renting MapReduce computing resources on demand. Puppet [20] is designed as an automation software that aids system administrators in managing and quickly deploying critical applications on large-scale servers. Cloudera Manager [21] is targeted for deploying Hadoop ecosystems for enterprise-class requirements, including additional enterprise management components and security enhancement packages. Apache Ambari [22] is designed to simplify Hadoop management. Although these tools and services are useful, some common functionalities required for using Hadoop computing clouds, hereafter referred to as Hadoop clouds, are not user-friendly for scientists without computer science expertise and relevant technical skills. Such examples include constructing a Hadoop cloud on idle computers of a laboratory and integrating bioinformatics MapReduce tools for a Hadoop cloud or users.

In this study, we present CloudDOE, a software package for deploying an on-demand computing cloud with minimal user intervention. CloudDOE integrates available MapReduce programs within a unified graphical interface, and extends their functions with the addition of new MapReduce programs. In addition, smart features are included in CloudDOE, e.g., an auto-configuring algorithm of the Deploy wizard and an isolation method of the Operate wizard. CloudDOE encapsulates the complicated and niggling procedures of manipulating a Hadoop cloud, and is hence suitable for users of MapReduce cloud computing tools.

## Results

**Figure 1:**
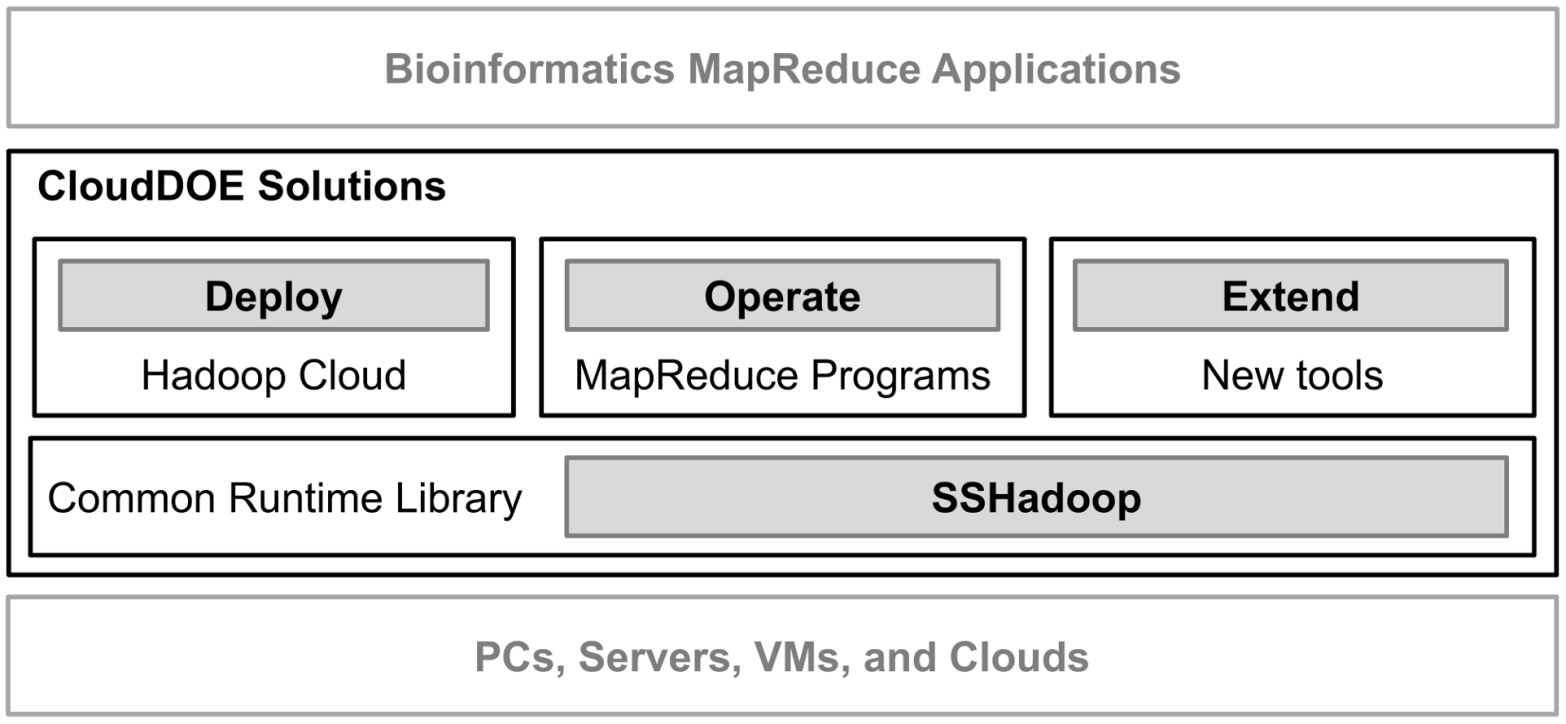
Software solutions of CloudDOE. A user can deploy a Hadoop Cloud, operate the supported bioinformatics MapReduce programs, and extend cloud functions through installing new tools.

**Figure 2:**
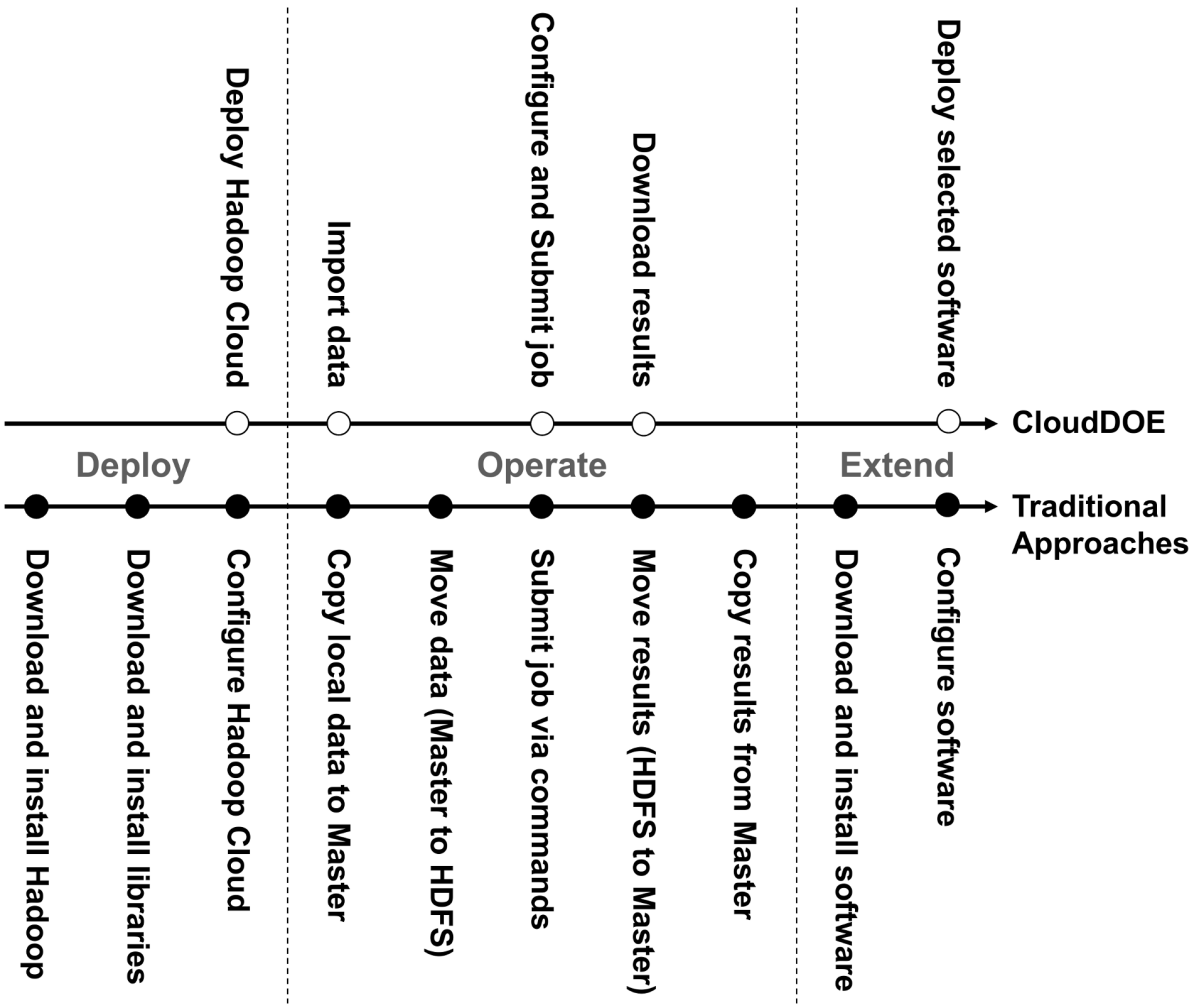
Comparison of CloudDOE and traditional approaches. CloudDOE encapsulates complicated procedures of traditional approaches into graphical user-friendly interfaces. Nearly 50% of the manipulating steps are reduced compared to traditional approaches.

CloudDOE aims at providing an open and integrated platform for biology/bioinformatics laboratories seeking to analyze big data via cloud computing with Hadoop/MapReduce (Figure 1). CloudDOE provides straightforward and user-friendly graphical interfaces, and covers most of the complicated, technical, and difficult command-line operations a user may encounter in traditional approaches (Figure 2). Several MapReduce programs are currently integrated into CloudDOE (Table 1). Since CloudDOE is implemented in Java, users can run it on various operating systems, e.g., Windows, Linux, and Mac OS, with Java runtime environments installed. Prerequisites of CloudDOE are provided in Supplementary section 1 of File S1.

**Table 1:** Currently integrated MapReduce programs.

Name	Description
CloudBurst	Highly sensitive short read mapping.
CloudBrush	A *de novo* genome assembler.
CloudRS	An error corrector of substitution sequencing.
Hadoop-Examples	Hadoop example applications, including WordCount and Grep programs. The streaming mode of WordCount program is also available.

### Deploying a Hadoop Computing Cloud

The Hadoop cloud deployment procedure involves installing runtime environments and configuring system parameters. A Java runtime environment and Hadoop distributions are the basic requirements for constructing a Hadoop cloud. To improve usability and to simplify the installation processes, we developed Deploy wizard, which guides users to build their own Hadoop cloud in only three steps. Users simply need to provide user credentials and network connection settings of each computer upon installation of the cloud. Thus, the otherwise complicated installation procedure is completed automatically.

Configuring a functional Hadoop cloud requires a computer science background and relevant operating skills, since improper parameter settings may affect cloud performance and incorrect system settings may lead to a malfunctioning system. To minimize the complexity of configuring a Hadoop cloud, we designed an auto-configuring algorithm in the Deploy wizard. The algorithm generates Secure Shell (SSH) certificates for internal communication and a set of cloud settings. This information is stored in files distributed to the cloud nodes as well as in the local PC for further use, e.g., for modifying cloud settings and re-deploying the cloud.

**Figure 3:**
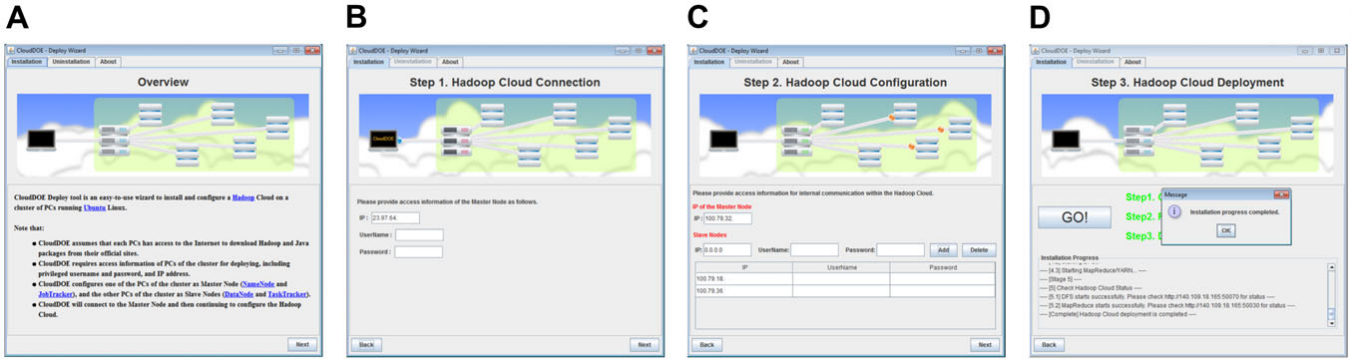
Screenshots of Deploy wizard. (A) Brief instructions to explain the system requirements and procedures that Deploy wizard will perform. A user is prompted (B) to provide information of the connection between the local PC and the Hadoop cloud and (C) to set up information of the Hadoop cloud, including IP addresses and a username/password. (D) Settings and configurations of the target cloud are generated automatically. The installation progress and logs can also be monitored on the wizard.

A Hadoop cloud consists of a master node and multiple slave nodes (Figure 3A). A user is prompted to fill in the IP address and user account/password for the master node (Figure 3B) and each slave node (Figure 3C). The deployment process often takes 10–15 minutes (Figure 3D). Users can also undeploy a Hadoop cloud installed by CloudDOE, and restore configurations using the uninstallation function of Deploy wizard. To understand the process quickly, users can watch the supplementary video of Deploy wizard for step-by-step instructions and useful tips (File S2).

In addition, CloudDOE is applicable for use in multiple deployment environments, e.g., hybrid and private/public clouds. An in-depth discussion of Deploy wizard is provided in Supplementary section 2 of File S1, including a list of necessary service ports used by Hadoop services and CloudDOE (Table S1 in File S1), an example of simulated machine information of a hybrid cloud on Windows Azure [23] (Figure S1 and Table S2 in File S1), and a list of files and directories affected during deployment (Table S3 in File S1). Advanced users can also download the development branches or manually change the configuration for deploying a Hadoop cloud with different Hadoop releases (Table S4 in File S1).

### Operating with Existing MapReduce Programs

**Figure 4:**
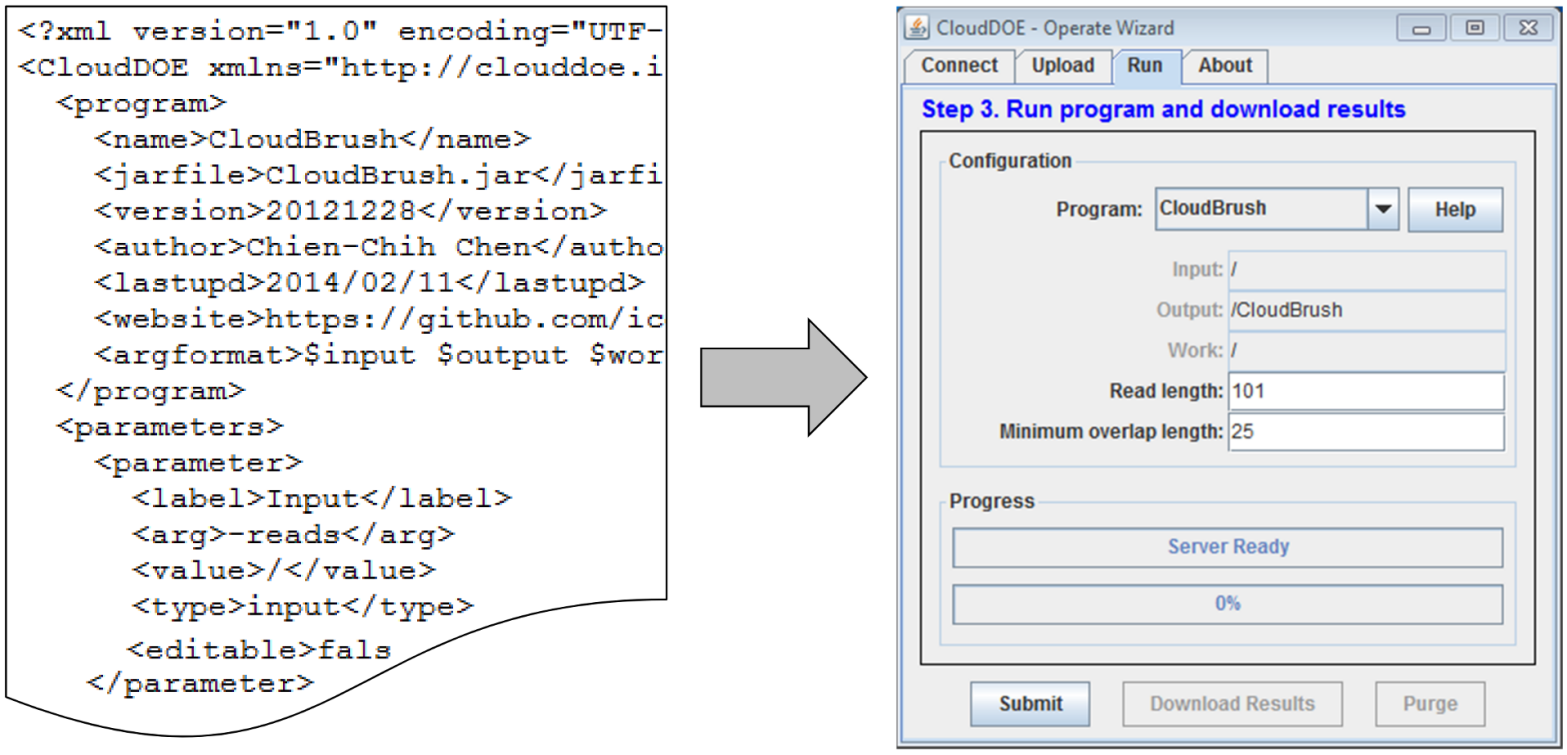
A structured XML configuration file and the generated wizard. The configuration file contains a metadata section on general program information, a set of parameters and its default values that are necessary to execute the program, and sections on log files and result download methods. CloudDOE loads a configuration file and generates the specific wizard required.

Several NGS data analysis tools have been implemented on the MapReduce framework. To overcome the hurdle of manipulating a MapReduce program with complicated command-line interfaces, we proposed a graphical wizard dubbed Operate. Users can manipulate a program with customized interfaces generated from necessary information in a configuration file, which is composed by the program’s author or an advanced user (Figure 4). An isolation method is also introduced to create a dedicated workspace for storing experimental data, i.e., programs, input files, and experimental results, of each execution. With Operate wizard, users can benefit from (1) a graphical interface for the MapReduce program, (2) a streamlined method for manipulating input/output data and setting up program parameters, and (3) a status tracker and progress monitor for execution.

**Figure 5:**
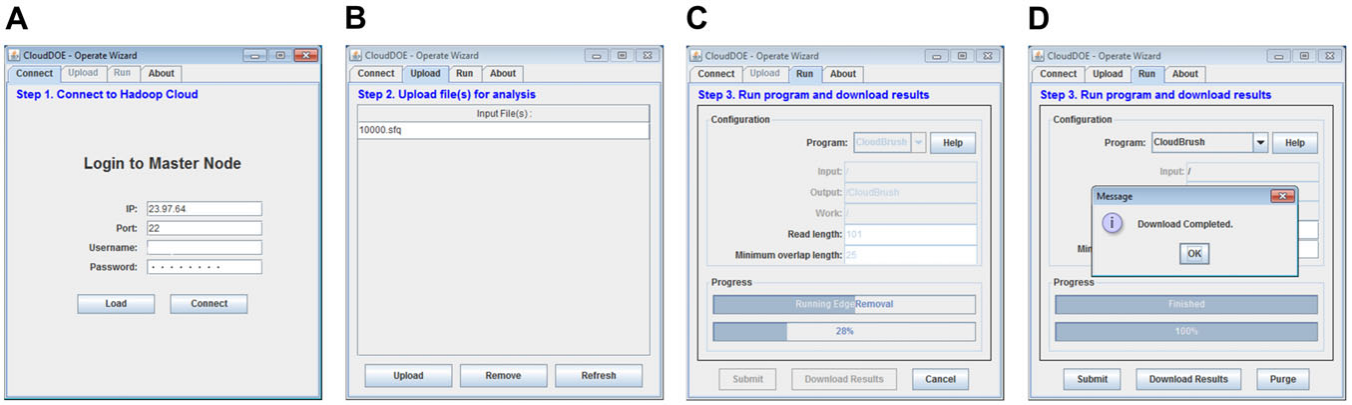
Screenshots of Operate wizard. A user can (A) log in to their Hadoop cloud, (B) upload and manage input data, (C) configure program parameters, and thus submit and monitor an execution, and (D) download the results after execution is completed.

The user can fill out or load the stored login information to log in to the Master node of a Hadoop cloud (Figure 5A). After a successful login, the user can upload data files to the Hadoop cloud (Figure 5B), select supported MapReduce programs, and specify parameters for execution (Figure 5C). We also designed two progress bars for monitoring the execution progress of the ongoing MapReduce step and the entire program. After the program execution is completed, the user can download experimental results to a local computer for further processing (Figure 5D). To understand the process quickly, users can watch the supplementary video of Operate wizard for step-by-step instruction and useful tips (File S3).

In addition, the tool-adding process of CloudDOE, which requires the MapReduce jar files and their configuration files in the same directory under the target Hadoop cloud, could only be carried out by advanced users. To further simplify the burden of adding tools to CloudDOE, we presented Extend wizard, which is an extension management center of a Hadoop cloud (Figure S2 in File S1). Note that the Extend wizard is currently a prototype, and detailed information is provided in Supplementary section 3 of File S1.

## Discussion

Hadoop/MapReduce supports large-scale computing in a distributed parallel and robust manner, thus ushering in a new era of bioinformatics data analysis. More bioinformatics tools are adopting the Hadoop/MapReduce framework. However, there are only a few software packages that currently provide bottom-tier support of MapReduce applications for general audiences, including developers, system administrators, and users. We thus developed CloudDOE, which provides cross-platform and user-friendly graphical interfaces, allowing a wider user base to manipulate a Hadoop cloud.

### Strengths and Limitations

CloudDOE is suitable as a unified console to Hadoop clouds among various computing environments, e.g., an in-house private cloud or rented machines from public cloud providers. CloudDOE is also useful and applicable across different scenarios: (1) deploying a workable Hadoop cloud with the auto-configuring algorithm within three steps, (2) manipulating a supported MapReduce program with the isolation method, and (3) integrating a MapReduce program with the program configuration file.

There are nonetheless several limitations of the current CloudDOE release. The auto-configuring algorithm is performed sequentially, and only supports Ubuntu Linux distribution. Program integration does not support constructing pipelines for multiple programs. The deploy function only supports deploying the most common Hadoop releases on machines without Hadoop-integrated environments (Table 2). Note that the support of deploying Apache Hadoop version 2 is released as a development branch of CloudDOE.

**Table 2:** Supports of CloudDOE for various deployment environments.

Deployment Environments				
Category	Examples	Hadoop Installed?	Hadoop Configured?	Supported by CloudDOE#?
Generic machines	PCs, servers, or VMs	No	No	Yes
Cloud providers	Amazon EC2	No	No	Yes
	RackSpace			
	Microsoft Azure			
	Hinet hicloud			
Hadoop-integrated environments	Cloud BioLinux	Yes	No	No

# CloudDOE supports deploying Apache Hadoop version 0.20.203 in the current release. The supports of deploying Apache Hadoop 1.2.1 and 2.2.0 are released as development branches.

### Comparison with Similar Deploying Tools

Several existing projects aim at easing the burden of deploying and managing a Hadoop cloud. Table 3 shows a comparison of the main features of current projects. The hicloud-hadoop, Apache Whirr, and Puppet projects are based on command-line interface, whereas the Cloudera manger, Apache Ambari, and CloudDOE projects provide graphical user interfaces. Apache Whirr supports deploying a Hadoop cloud through composing proper deployment files, thus initiating machine instances from infrastructure-as-a-service providers. Puppet supplies functions for deploying, enhancing, and managing a Hadoop cloud through executing appropriate modules developed by experts. Cloudera Manager and Apache Ambari provide functions for manipulating a Hadoop cloud. However, computer science expertise is still necessary to accomplish technical operations, e.g., generate and exchange SSH key pairs and adapt system configuration files. CloudDOE presents functions for deploying and undeploying a Hadoop cloud for administrators, and encapsulates technical operations using wizards. It also supports the manipulation of available bioinformatics MapReduce programs on a Hadoop cloud for a bioinformatician.

**Table 3:** Features comparison of CloudDOE and similar deploying tools.

	Function#				
Project	Deploy	Operate	Extend	User Interface#	IaaS Only?#
hicloud-hadoop	√	N/A	N/A	CLI	No
Apache Whirr	√	N/A	N/A	CLI	Yes
Puppet	√	N/A	√	CLI	No
Cloudera Manager	√	N/A	√	GUI (Web)	No
Apache Ambari	√	N/A	√	GUI (Web)	No
CloudDOE	√	√	√	GUI (Java)	No

# N/A, not available in the current release of the project; IaaS, infrastructure-as-a-service; CLI, command-line interface; GUI, graphical-user interface.

### Future Work

Parallel processing utilizes non-blocking operations and job overlapping to reduce waiting latency, and has been applied to different situations. We would like to accelerate deploying progress by introducing a parallel dispatcher and a monitor mechanism in future CloudDOE releases. One of the most successful characteristics of the existing workflow or execution platforms is the ability for users to construct analysis pipelines from available programs. Thus, incorporating the MapReduce programs into a pipeline with stand-alone programs to replace time-consuming processes is a promising future direction. We plan to implement wrapper functions or tools to integrate the MapReduce programs into workflows of existing bioinformatics platforms, e.g., Galaxy [24]. To enhance and keep up with technology trends, we plan to support state-of-the-art big data computing platforms, e.g., Hadoop BigTop [25] and Spark [26]. We also welcome community efforts to collaborate in future developments and in the maintenance of CloudDOE for integrating more MapReduce bioinformatics tools, providing multivariate deploying environment support, e.g., Cloud BioLinux [27], and supporting next-generation big data open source tools.

## Conclusion

We have presented CloudDOE, a software package with user-friendly graphical wizards. CloudDOE supports users without an advanced computer science background in manipulating a Hadoop cloud, and thus reduces operation costs by encapsulating technical details and niggling command-line processes. CloudDOE also improves the usability of existing bioinformatics MapReduce programs by integrating these programs into a unified graphical user interface. We have also demonstrated that CloudDOE is useful and applicable for different scenarios and targeted users, including ordinary users, developers, and administrators. CloudDOE is an open-source project distributed under Apache License 2.0 and is freely available online.

## Materials and Methods

**Figure 6:**
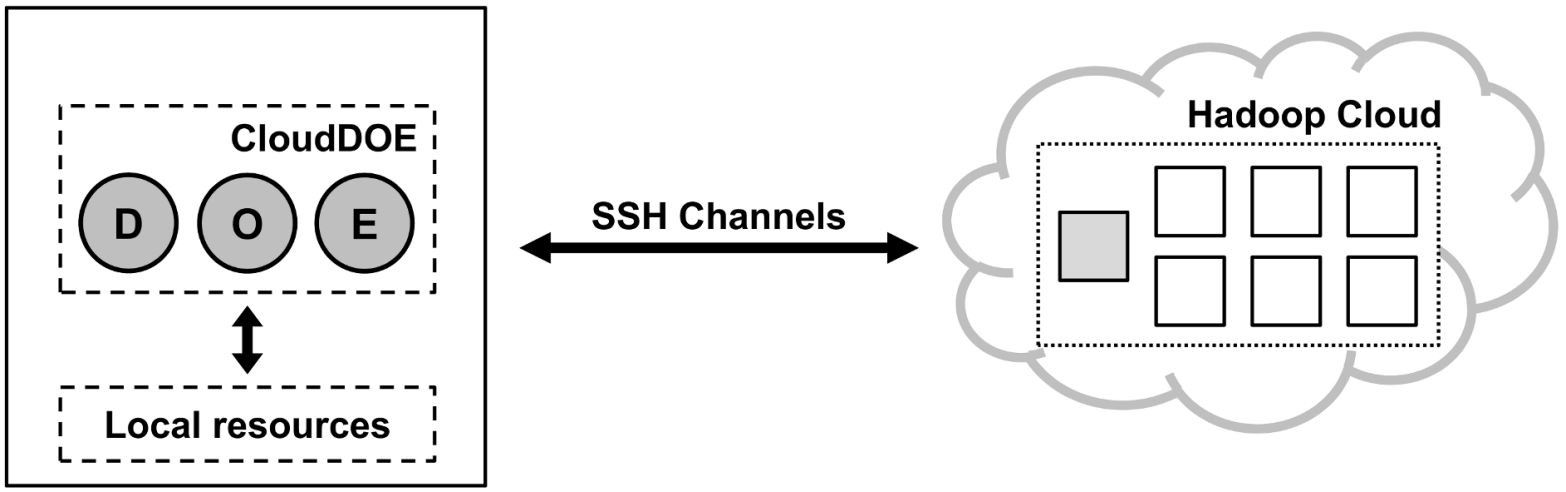
System architecture of CloudDOE. The solid square represents a machine or a computing resource, and the gray solid square is the master of the Hadoop cloud. CloudDOE establishes Secure Shell (SSH) channels for communication and acquires local resources for operations.

To operate a Hadoop cloud remotely, we employed the client-server model as the system architecture of CloudDOE. Client-side applications were developed by Java and encapsulated as Java archive (JAR) executable files designed to be executed smoothly across different operating systems and environments, e.g., Windows, Linux, and Mac OS. Server-side deploying agents were written in GNU Bourne-Again Shell (BASH) script language because of its flexibility and good support for most Linux distributions. Figure 6 shows the system architecture of CloudDOE. Further details about the interaction of CloudDOE and a Hadoop cloud are provided in Supplementary section 4 of File S1, including the Deploy, Extend (Figure S3 in File S1), and Operate (Figure S4 in File S1) wizards.

Communications between clients and the server were conducted through SSH channels in a reliable and secure manner. SSH is a cryptographic network protocol that aims to communicate securely within an insecure network environment. We developed SSHadoop, a library inherited from JSch [28], to establish secure communication channels and execute commands. It also enables the ability to complete basic demands and operations of CloudDOE, including remote program execution and job monitoring, data import and management, and downloading of experimental results.

An auto-configuring algorithm was a major component necessary for deploying a Hadoop cloud from CloudDOE. Runtime environments and dependent libraries were installed through our server-side agents, currently applied to the Ubuntu Linux distribution. A set of security credentials (e.g., SSH key pair) was generated for internal usage, e.g., communication and services control, for configuring a Hadoop cloud. Moreover, pre-formatting cloud settings were also produced and applied globally. The configuration files contain role types of a Hadoop cloud in each computer (i.e., master or slave), a number of data replicas and relevant configurations of Hadoop Distributed File System, and operating system-related settings.

A unique isolation identifier (IID) was the core concept of the isolation method, which is aimed at constructing independent workspaces and distinguishing the operation scope of executions. An IID is composed of a magic number and a user identifier, i.e., a timestamp followed by the current username. It is generated and applied to Hadoop cloud the first time an integrated program is initiated. We also exploited the IID to implement a stateful interaction environment, e.g., execution status recovery, to improve the reliability of connection and usability.

A structured extensible markup language (XML) configuration file was utilized to integrate a MapReduce program into CloudDOE. This XML file is composed of various information blocks, i.e., program, parameters, logs, and downloads (Figure S5 in File S1). The *program* block expresses general information of the program. In the *parameters* block, parameters and their default values are defined. The *logs* block lists a program log file provided by authors that can be used to monitor program execution. Output files and their corresponding download methods are defined in the *downloads* block. Detailed information of each configuration field is given in Supplementary section 5 of File S1 for interested users.

## Supporting Information

File S1 **Supplementary information, figures, and tables.** Figure S1. A Hadoop cloud environment simulated from real Microsoft Azure machine data. Figure S2. Screenshots of Extend wizard. Figure S3. Interactions between CloudDOE, Hadoop cloud and Internet when manipulating a Hadoop cloud with Deploy or Extend function. Figure S4. Interactions between CloudDOE and Hadoop cloud when manipulating a Hadoop cloud with Operate function. Figure S5. Format of the program integration configuration file of CloudDOE.
(PDF)

File S2 **A step-by-step video of Deploy wizard with useful tips.**
(MP4)

File S3 **A step-by-step video of Operate wizard with useful tips.**
(MP4)
