# Supplementary material for: CloudDOE: A User-Friendly Tool for Deploying Hadoop Clouds and Analyzing High-Throughput Sequencing Data with MapReduce
Source: PLoS One. 2014 Jun 4;9(6):e98146. doi: 10.1371/journal.pone.0098146 (PMC4045712; doi:10.1371/journal.pone.0098146)
Supplement: File S1 — Supplementary information, figures, and tables. Figure S1. A Hadoop cloud environment simulated from real Microsoft Azure machine data. Figure S2. Screenshots of Extend wizard. Figure S3. Interactions between CloudDOE, Hadoop cloud and Internet when manipulating a Hadoop cloud with Deploy or Extend function. Figure S4. Interactions between CloudDOE and Hadoop cloud when manipulating a Hadoop cloud with Operate function. Figure S5. Format of the program integration configuration file of CloudDOE. (PDF) [file pone.0098146.s001.pdf]

File S1: Supplementary Information of CloudDOE

Table of Contents

1. Prerequisites of CloudDOE....2
2. An In-depth Discussion of Deploying a Hadoop Cloud....2
    Prerequisites of deployment....2
    Table S1. List of necessary service ports used by Hadoop services and CloudDOE..3
    Table S2. Example of machine information and Hadoop cloud settings....3
    Figure S1. A Hadoop cloud environment simulated from real Microsoft Azure machine data. 4
    Management of configuration changes....4
    Table S3. List of files and directories affected by CloudDOE....4
    Methods to deploy a Hadoop cloud with different release....4
    Table S4. List of Hadoop resources configuration used by CloudDOE. 5
3. A Prototype for Extending the Functionalities of a Hadoop Cloud....5
    Figure S2. Screenshots of Extend wizard. 5
    The extension management center of Extend Wizard....6
4. Interactions between CloudDOE Components....6
    Figure S3. Interactions between CloudDOE, Hadoop cloud and Internet when manipulating a Hadoop cloud with Deploy or Extend function. 7
    Figure S4. Interactions between CloudDOE and Hadoop cloud when manipulating a Hadoop cloud with Operate function. 7
5. Schema of the Program Integration Files....7
    Figure S5. Format of the program integration configuration file of CloudDOE. 9
References....10

1. Prerequisites of CloudDOE

CloudDOE is an open source project and it is announced as Apache license 2.0. It installs Java runtime environment version 1.6 and deploys a Hadoop cloud with Apache Hadoop [1] version 0.20.203. Binaries and source code of CloudDOE are hosting on website (http://clouddoe.iis.sinica.edu.tw/) and GitHub (https://github.com/moneycat/CloudDOE). Demo videos are also available on the website, and provided as supplementary materials of the CloudDOE paper. The following are steps to install and run CloudDOE.

(1) Make sure Java runtime environment (JRE) version 1.6 or later are installed on the local computer that you are going to run CloudDOE.
(2) Download and decompress the latest CloudDOE binary archive into the local computer.
(3) Run Deploy.jar located in the local directory of CloudDOE to deploy a Hadoop cloud on those Ubuntu Linux computers.
(4) Run Operate.jar located in the local directory of CloudDOE for MapReduce programs, e.g., CloudBurst, CloudRS and CloudBrush.

For interested developers, building CloudDOE from source code involves different requirements as follows.

(1) Java development kit (JDK) and JavaFX runtime environment (jfxrt) are required. JDK version 1.7 or later is encouraged for better support of successfully building CloudDOE for users, since jfxrt is evolved in the JDK release. Otherwise, users should download the correct version of jfxrt package from Oracle and integrate the package into development environment manually.
(2) Integrated development environment (IDE) tools, e.g., Eclipse, may be useful for building CloudDOE. Users can import source code into the IDE, or utilizing Java native command-line tools, i.e., javac and jar, to build CloudDOE.

2. An In-depth Discussion of Deploying a Hadoop Cloud

Prerequisites of deployment

Users need to prepare computers for Hadoop cloud building before running CloudDOE. A Hadoop cloud deployed with CloudDOE need at least two computers, i.e., one for the master and the others for slaves, for better performance comparing to a local mode Hadoop environment. Users need to A) prepare two or more Ubuntu Linux computers to build a Hadoop cloud, and B) collect IP addresses and super-user accounts for those Ubuntu Linux computers before running CloudDOE.

For computers protected by advanced firewall settings, there are several prerequisites to ensure proper function and correct manipulation of a Hadoop cloud. Table S1 lists service-dependent ports required by Hadoop services on Transmission Control Protocol (TCP) layer. Port 22 is used to establish Secure Shell (SSH) channels for the Hadoop

cloud and CloudDOE. Port 9000 and 9001 are custom defined endpoints for Hadoop Distributed File System (DFS) and MapReduce, respectively. The rest of the ports are default interfaces of Hadoop services.

Table S1. List of necessary service ports used by Hadoop services and CloudDOE.

TCP Port	*Master*		*Slave*		*Description*
	Name Node	*Job Tracker*	*Data Node*	*Task Tracker*	
22	V	V	V	V	SSH endpoint.
9000	V				DFS endpoint.
9001		V			MapReduce endpoint.
50010	V		V		DFS Data transfer channel.
50020	V		V		DFS Inter-Process Communication.
50030		V			Web/connection interfaces.
50060				V	Web/connection interfaces.
50070	V				Web/connection interfaces.
50075			V		Web/connection interfaces.

Machine information is necessary for deploying a Hadoop cloud, and can be obtained from cloud service providers or system administrators when requesting computing resources. Table S2 lists necessary information for deploying a Hadoop cloud, and lists associated Hadoop role types assigned for each machine by CloudDOE. In Table S2, firewall and network connection information are simulated from real Microsoft Azure [2] virtual machine data. This simulated data forms a general environment of a private (or hybrid) cloud since machines are partially under a network address translation (NAT) layer as shown in Figure S1. In Table S2, the external Internet protocol (IP) address of machine A is used for communication between CloudDOE and a Hadoop cloud, whereas communications within Hadoop services are through internal IP addresses. In this scenario, users must contact service providers or system administrators for assistance before deploying a Hadoop cloud to prevent the necessary service ports are blocked by firewalls.

Table S2. Example of machine information and Hadoop cloud settings.

ID	*User Credential (username/password)*	*Network Connection*		*Firewall Enabled*	*Hadoop Role Types*	
		External IP	*Internal IP*		*Master*	*Slave*
A	clouddoe/********	23.97.64.xxx	100.79.32.71	Yes	V	V
B	clouddoe/********	N/A	100.79.18.66	Yes		V
C	clouddoe/********	N/A	100.79.36.13	Yes		V

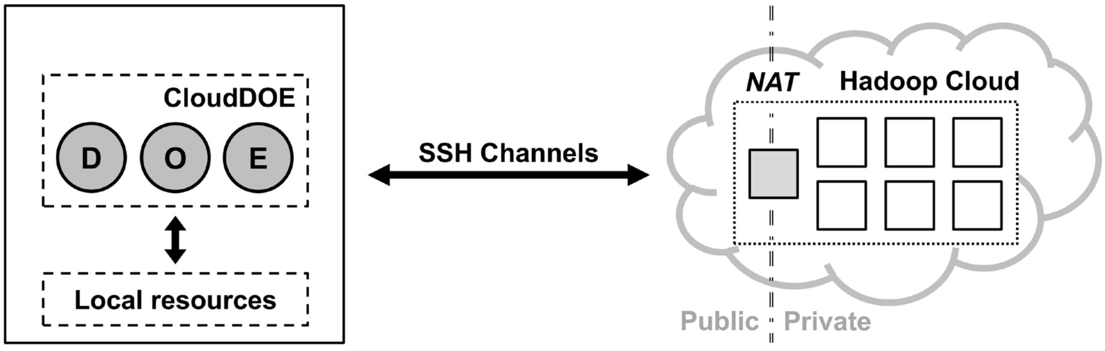

Figure S1. A Hadoop cloud environment simulated from real Microsoft Azure machine data. A solid square is a machine or a computing resource, and a gray solid square is the master of the Hadoop cloud. It is a hybrid cloud where the slaves are in a private network and the master owns public network access rights.

Management of configuration changes

CloudDOE creates and modifies necessary files of a Hadoop cloud during deployment. Since the files may only be accessed by administrators, accounts with super-user privileges, i.e., users in the wheel group, are encouraged to deploy the Hadoop cloud. Table S3 lists files and directories CloudDOE may affect.

Table S3. List of files and directories affected by CloudDOE.

*File or Directory Location**	*Description*
/etc/hostname	Identity of the computer in the Hadoop cloud.
/etc/hosts	Static resolution for mapping computer identities.
/opt/hadoop	Directory of Hadoop MapReduce installation.
/var/hadoop	Directory of Hadoop MapReduce and DFS runtime environment.
$HOME/.ssh	Directory of security credentials used for internal connection.
$HOME/workspace	Directory of CloudDOE.

* The *$HOME* variable indicates the home directory of a user account.

The original configuration files are renamed with a timestamp and saved in the same directory for each installation by CloudDOE. The saved files are used to restore the system to the state before deployment. An interested user can achieve the deploying history through consoles.

Methods to deploy a Hadoop cloud with different release

CloudDOE downloads and installs Apache Hadoop version 0.20.203 by default. An interested user can download the development branches, or manually change the Hadoop release configuration to deploy a cloud with different Hadoop releases. Table S4 lists the configurations are located in the file at *workspace/bin/32_install_hadoop.sh*.

In the current CloudDOE release, the deploying function has been investigated with most common Apache Hadoop releases, i.e., version 0.20.203 and 1.2.1. The support of

deploying Hadoop version 2 is also released as a development branch. We wish interested users and communities can join us to develop and examine the deployment procedure with different Hadoop distributions, e.g., Cloudera CDH [3], Hortonworks [4] and MAPR Hadoop [5], and releases.

Table S4. List of Hadoop resources configuration used by CloudDOE.

Variable	*Default Value*	*Description*
HADOOP_WWW	http://archive.apache.org/dist	Target download location
HADOOP_VER	hadoop-0.20.203.0	Target Hadoop version
HADOOP_TAR	rc1.tar.gz	Suffix of target resource file

3. A Prototype for Extending the Functionalities of a Hadoop Cloud

The functionalities of a Hadoop cloud are greatly enhanced by installing additional tools or platforms. For example, Hadoop related projects extend the ability of cloud by forming a distributed database [6, 7] and accomplishing ad-hoc queries [8], or providing a high-level platform and its correspond language for creating MapReduce programs [9]. Since the functionalities are value added for bioinformatics [10, 11], the installation procedure also needs specific knowledge similar to deploy a Hadoop cloud. To reduce the obstacles encountered in enhancement, we built Extend wizard, an extension management center of a Hadoop cloud. Extend wizard is designed as a dashboard of collected cloud computing plug-ins, e.g., tools and platforms. It provides an approach for users to install plug-ins onto a Hadoop cloud. We demonstrated the features of Extend wizard with Cloudgene [12], a web-based MapReduce execution platform for bioinformatics. In conclusion, users can browse and install plug-ins, and monitor the installation progress through the wizard. Furthermore, program providers and developers can also incorporate their programs into our management center by writing suitable installation scripts. Figure S2 shows screenshots of Extend wizard.

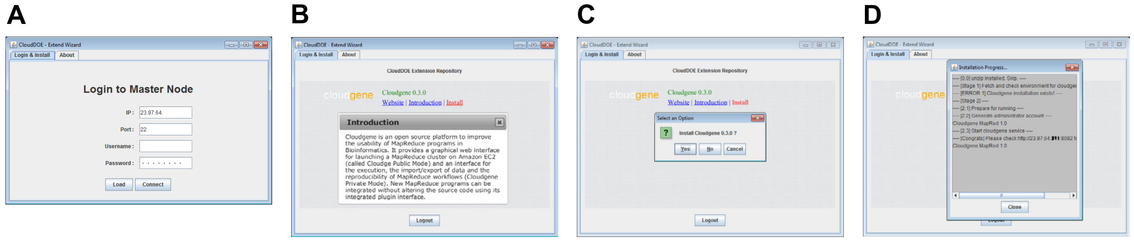

Figure S2. Screenshots of Extend wizard. (A) User login to their Hadoop Cloud first. (B) The Extension Management dashboard provides a way for users to select and install supported applications to their Cloud. (C) A confirm window for the installation procedure. (D) Users can also monitor the installation procedures.

Because the demand of new MapReduce tools of each laboratory is different, it requires community efforts to maintain a repository of MapReduce tools and Hadoop-related add-

ons with metadata and installation scripts. We will continue to provide fundamental supports toward this aim in Extend wizard.

The extension management center of Extend Wizard

The extension management center embedded a web browser component to handle interactions between a user and the cloud. Thus, HTML and JavaScript libraries (i.e., jQuery and jQuery UI) are utilized to organize a web-based interactive dashboard. An installation request launched from JavaScript is translated to proper install information, including the location of installation scripts and technical parameters, and pass to server side agents through the extend wizard. Therefore, installation procedures are controlled by the agents, and users can monitor installation progresses with the same mechanisms of deployment.

4. Interactions between CloudDOE Components

Figure S3 and S4 show the iterations between CloudDOE, a Hadoop cloud and Internet when manipulating a Hadoop cloud through CloudDOE. The procedures of deploying or extending a cloud with CloudDOE have similar steps, which shown in Figure S3. Step 1 is the only one step that requires users to provide information for installation, i.e., IP address and user credentials of each computer. CloudDOE then uploads installation-related resources to the master automatically (Step 2), and initiates installation procedures (Step 3). Required software, i.e., Java and Hadoop distribution, is requested and downloaded from the Internet (Step 4) during installation. Users can monitor installation progress through CloudDOE (Step 5). Figure S4 demonstrates interactions of operating a MapReduce program with CloudDOE. There are 3 major steps for users to performing an execution after successfully connect to the master of a Hadoop cloud as follows. Step 1, import data to Hadoop cloud for analysis. Step 2 and 3 set up program parameters and submit the execution request to master of the Hadoop cloud. Users can monitor the progress during execution (Step 4), and download results from Hadoop cloud after successful execution (Step 5).

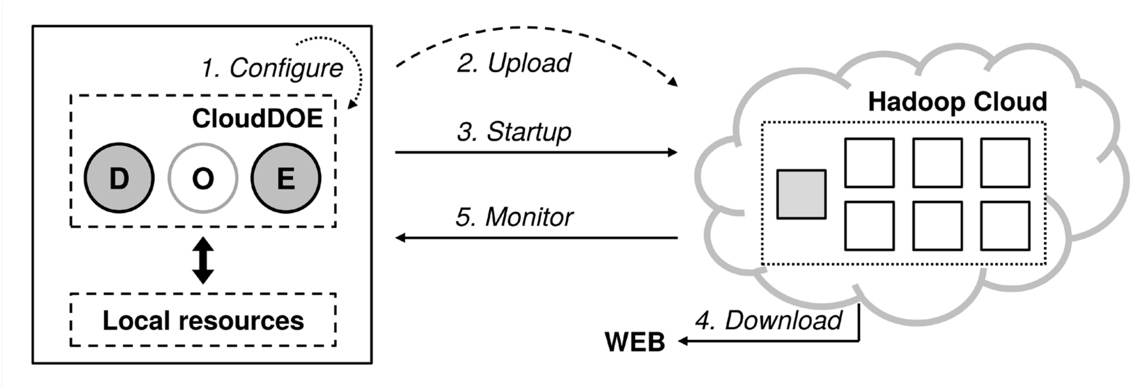

Figure S3. Interactions between CloudDOE, Hadoop cloud and Internet when manipulating a Hadoop cloud with Deploy or Extend function. A dashed arrow is the data flow and a dotted arrow is the control flow. A solid square is a machine or a computing resource, and a gray solid square is the master of the Hadoop cloud. CloudDOE requires 4 steps to complete a request. Users only have to complete the first step, and thus, the rest of steps are initiated and controlled by CloudDOE.

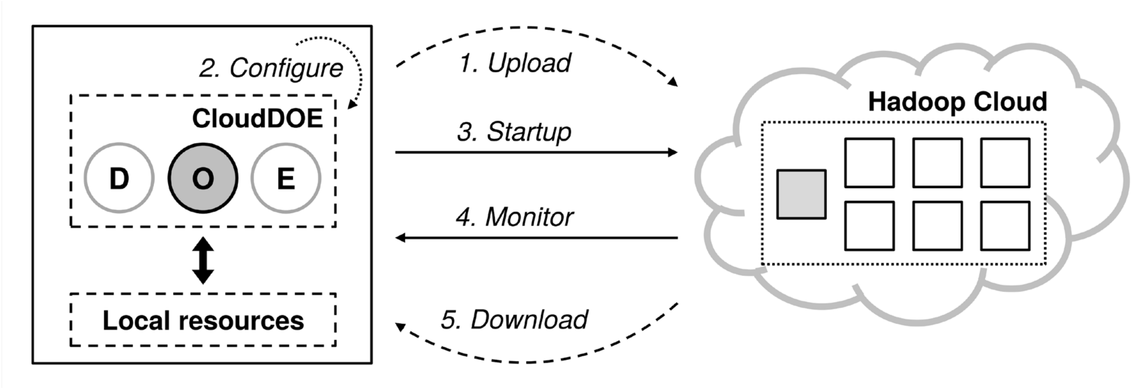

Figure S4. Interactions between CloudDOE and Hadoop cloud when manipulating a Hadoop cloud with Operate function. A dashed arrow is the data flow and a dotted arrow is the control flow. A solid square is a machine or a computing resource, and a gray solid square is the master of the Hadoop cloud. To operate a MapReduce program through CloudDOE, users need to upload data for analysis, configure program parameters, and then submit the execution request to the Hadoop cloud. Users can also monitor execution progress and download results from the Hadoop cloud.

5. Schema of the Program Integration Files

CloudDOE utilizes a structured extensible markup language (XML) file for program integration. A configuration file consists of 4 main blocks, i.e., *program*, *parameters*, *logs*, and *downloads*. There are also multiple configuration fields or blocks contained in each main block, as shown in Figure S5, which is described as follows.

(1) The *program* block contains general program information.

- The <*name*>, <*author*>, <*version*>, and <*website*> fields indicate the program name, author of the program, program release version, and website respectively.
- The <*jarfile*> field specifies the file name of the target MapReduce file.
- The <*streaming*> is an optional field for streaming mode. CloudDOE may execute the target program in streaming mode if the value is *True*.
- The <*lastupd*> field contains the last update timestamp of this configuration file.
- The <*argformat*> is the most important field which defined the format of argument list used by the target program. Each argument in the format list represent as a variable, e.g., *$input*, *$output*, *$work*, and *$value*, which is mapped to the <*type*> field in each *parameter*. CloudDOE fills the variables and passes them to the target program.

(2) The *parameters* block contains multiple *parameter* blocks. A valid CloudDOE integration configuration file must contains at least one *input*, *out*, and *work* parameter for a target program. A *parameter* block consists of the following fields.

- The <*label*> field represents the display label of the parameter input area on CloudDOE interface.
- The <*editable*> field controls whether the parameter input area can be edited by users or not.
- The <*arg*> and <*value*> fields are the argument name and its default value of the target program.
- The <*type*> field indicates the parameter type used by CloudDOE. There are various values of the field: (a) *input*, *output* and *work* are required fields for a program, (b) *mapper*, *reducer*, and *file* are used when the execution mode is streaming, and (c) a set of *value* represents the other parameters.

(3) The *logs* block may contain multiple *log* blocks. A log is produced by the standard output of the program, and is used to monitor the execution. It can also be downloaded for further analysis. A log field is composed by the following fields.

- The <*name*> field is the log file name generated by the target program. CloudDOE uses the value of the *work* parameter as prefix of the log file, and thus, perform further monitor processes.
- The <type> field is used to determine the major log when there are multiple log files pf the target program.

(4) The *downloads* block may contain multiple *download* blocks. A *download* block is corresponding to a result produced by the target program. Each download block is consisted of the following fields.

- The <*src*> field is the name of target result. The target result may be a field or a directory contains a bunch of files. CloudDOE uses the value of the *output* parameter as its parent directory.

- The <*dst*> field represents the name used to stored the result in local computer.
- The <*merge*> is an optional field that indicates the download method. The target result will be merged into a single file if the value is *True*.

Since composing a valid CloudDOE program integration configuration of a MapReduce program may still be a complicated work to authors, we wish interested users or communities can join us to improvement the integration method.

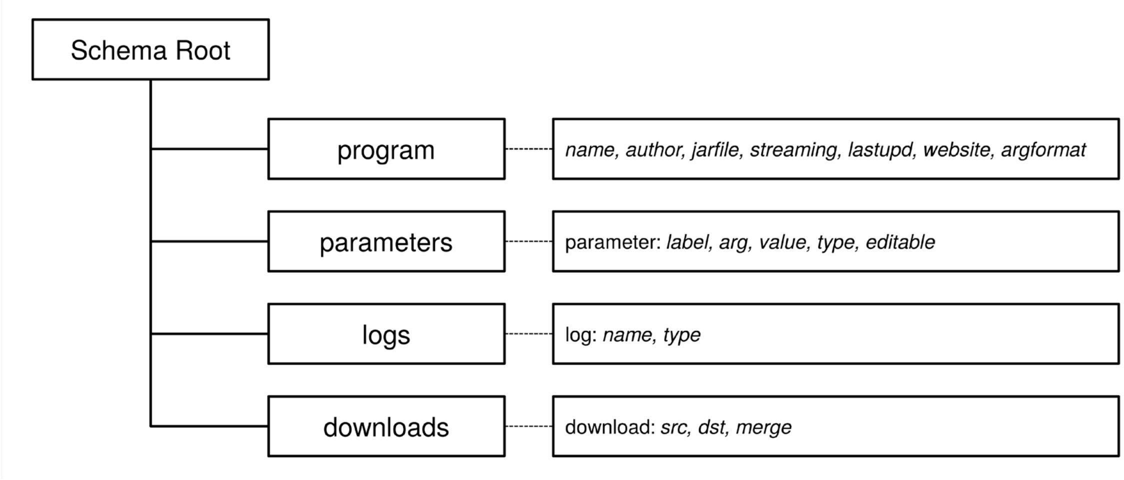

Figure S5. Format of the program integration configuration file of CloudDOE. A configuration file is defined in extensible markup language (XML), and contains 4 main blocks, i.e., program, parameters, logs, and downloads. There are multiple configuration fields or blocks in each main block.

References

1. Apache. Welcome to Apache™ Hadoop®! Available: http://hadoop.apache.org/.
2. Microsoft. Windows Azure. Available: http://www.windowsazure.com/.
3. Cloudera. Cloudera CDH. Available: http://www.cloudera.com/content/cloudera/en/products-and-services/cdh.html.
4. Hortonworks. MapReduce. Available: http://hortonworks.com/hadoop/mapreduce/.
5. MAPR. Hadoop. Available: http://www.mapr.com/.
6. Apache. Apache HBase™. Available: http://hbase.apache.org/.
7. Apache. Apache Cassandra. Available: http://cassandra.apache.org/.
8. Apache. Welcome to Apache Hive™! Available: http://hive.apache.org/.
9. Apache. Welcome to Apache Pig! Available: https://pig.apache.org/.
10. Nordberg H, Bhatia K, Wang K and Wang Z (2013) BioPig: a Hadoop-based analytic toolkit for large-scale sequence data. Bioinformatics 29: 3014-3019.
11. Schumacher A, Pireddu L, Niemenmaa M, Kallio A, Korpelainen E, et al. (2014) SeqPig: simple and scalable scripting for large sequencing data sets in Hadoop. Bioinformatics 30: 119-120.
12. Schonherr S, Forer L, Weissensteiner H, Kronenberg F, Specht G, et al. (2012) Cloudgene: a graphical execution platform for MapReduce programs on private and public clouds. BMC bioinformatics 13: 200.
